# Relationship of Metabolic Dysfunction-Associated Steatohepatitis-Related Hepatocellular Carcinoma with Oral and Intestinal Microbiota: A Cross-Sectional Pilot Study

**DOI:** 10.3390/medicina60071150

**Published:** 2024-07-17

**Authors:** Takaaki Matsui, Toshiya Morozumi, Yuko Yamamoto, Takashi Kobayashi, Ryo Takuma, Masato Yoneda, Asako Nogami, Takaomi Kessoku, Muneaki Tamura, Yoshiaki Nomura, Toru Takahashi, Yohei Kamata, Shuntaro Sugihara, Kyoko Arai, Masato Minabe, Norio Aoyama, Kenji Mitsudo, Atsushi Nakajima, Motohiro Komaki

**Affiliations:** 1 Department of Periodontology, Faculty of Dentistry, Kanagawa Dental University, Yokosuka 238-8580, Japan; 2 Department of Endodontics, The Nippon Dental University School of Life Dentistry at Niigata, Niigata 951-8580, Japan; 3 Department of Dental Hygiene, Kanagawa Dental University, Junior College, Yokosuka 238-8580, Japan; 4 Department of Gastroenterology and Hepatology, Yokohama City University Graduate School of Medicine, Yokohama 236-0004, Japan; 5 Department of Palliative Medicine and Gastroenterology, International University of Health and Welfare, Narita Hospital, Narita 286-8520, Japan; 6 Department of Microbiology and Immunology, Nihon University School of Dentistry, Tokyo 101-8310, Japan; 7 Institute of Photochemistry and Photofunctional Materials, University of Shanghai for Science and Technology, Shanghai 200093, China; 8 Faculty of Pharmaceutical Sciences, Nihon Pharmaceutical University, Saitama 362-0806, Japan; 9 Department of Advanced Periodontology, Faculty of Dentistry, Kanagawa Dental University, Yokohama 221-0835, Japan; 10 Bunkyo-Dori Dental Clinic, Chiba 263-0024, Japan; 11 Department of Education Planning, Kanagawa Dental University, Yokosuka 238-8580, Japan; 12 Department of Oral and Maxillofacial Surgery, Yokohama City University Graduate School of Medicine, Yokohama 236-0004, Japan

**Keywords:** microbiota, oral bacteria, intestinal bacteria, hepatocellular cancer, metabolic dysfunction-associated steatohepatitis, *Porphyromonas gingivalis*

## Abstract

*Background and Objectives*: The incidence of metabolic dysfunction-associated steatohepatitis (MASH)-related hepatocellular carcinoma (HCC) is increasing worldwide, alongside the epidemic of obesity and metabolic syndrome. Based on preliminary reports regarding the potential association of HCC and periodontitis, this study aimed to analyze the involvement of periodontal bacteria as well as the oral and intestinal bacterial flora in MASH-related HCC (MASH-HCC). *Materials and Methods*: Forty-one patients with MASH and nineteen with MASH-HCC participated in the study, completing survey questionnaires, undergoing periodontal examinations, and providing samples of saliva, mouth-rinsed water, feces, and peripheral blood. The oral and fecal microbiome profiles were analyzed by 16S ribosomal RNA sequencing. Bayesian network analysis was used to analyze the causation between various factors, including MASH-HCC, examinations, and bacteria. *Results*: The genus *Fusobacterium* had a significantly higher occupancy rate (*p* = 0.002) in the intestinal microflora of the MASH-HCC group compared to the MASH group. However, *Butyricicoccus* (*p* = 0.022) and *Roseburia* (*p* < 0.05) had significantly lower occupancy rates. The Bayesian network analysis revealed the absence of periodontal pathogenic bacteria and enteric bacteria affecting HCC. However, HCC directly affected the periodontal bacterial species *Porphyromonas gingivalis*, *Tannerella forsythia, Fusobacterium nucleatum,* and *Prevotella intermedia* in the saliva, as well as the genera *Lactobacillus*, *Roseburia*, *Fusobacterium*, *Prevotella*, *Clostridium*, *Ruminococcus*, *Trabulsiella*, and SMB53 in the intestine. Furthermore, *P. gingivalis* in the oral cavity directly affected the genera *Lactobacillus* and *Streptococcus* in the intestine. *Conclusions*: MASH-HCC directly affects periodontal pathogenic and intestinal bacteria, and *P. gingivalis* may affect the intestinal bacteria associated with gastrointestinal cancer.

## 1. Introduction

Hepatocellular carcinoma (HCC) is one of the frequent malignancies and a leading cause of cancer-related deaths globally [1]. Most cases of HCC are caused by viral hepatitis; however, the incidence has gradually decreased due to the direct-acting antiviral drug therapy for hepatitis C and nucleic acid analog therapy for hepatitis B [2,3]. Conversely, the incidence of HCC (MASH-HCC) related to metabolic dysfunction-associated steatohepatitis (MASH, formerly known as NASH) [4] is increasing worldwide, alongside the epidemic of obesity and metabolic syndrome [5]. However, the pathogenesis of MASH-HCC remains unclear.

In the past, the mechanism by which periodontal bacteria affect the entire body was thought to be as follows: bacteria enter the bloodstream directly from periodontal pocket ulceration and affect the organs outside the oral cavity [6,7]. In recent studies, another mechanism has been identified: oral bacteria in saliva are transferred enterally to the intestine by swallowing and affect the intestinal bacterial flora and metabolism [8]. The liver is an organ of the digestive system that is anatomically and physiologically connected to the enterohepatic circulation via the portal vein. Hence, periodontal bacteria and the lipopolysaccharides (LPSs) derived from the periodontal bacteria in the saliva may be implicated in the pathogenic mechanism of MASH-HCC by affecting the intestinal flora.

Epidemiological reports have shown that periodontal pathogen is a risk factor in the onset of various cancers and cancer-associated mortality [9]. *Fusobacterium nucleatum* was specifically detected in the organs of the digestive system that are anatomically close to the oral cavity [10,11,12], and the presence of *Porphyromonas gingivalis* was correlated with the malignancy of esophageal cancer [13]. Only a few reports have shown an association between HCC and periodontal pathogens, such as high circulating reactive oxygen species levels in patients with HCC and periodontitis [14]. As far as we are aware, our recent report showing an association between MASH-HCC and salivary *P. gingivalis*, *F. nucleatum*, and immunoglobulin A (IgA) is the only report to date highlighting a relationship between MASH-HCC and periodontopathic bacteria [15].

Based on these reports, we hypothesized that MASH-HCC is associated with periodontopathic bacteria in the oral cavity. This study aimed to analyze the clinical parameters and oral and intestinal bacterial flora in patients with MASH and MASH-HCC to determine the relationship between MASH-HCC and periodontal bacteria.

## 2. Materials and Methods

### 2.1. Participants

The participants in this study included patients with MASH and MASH-HCC aged 20 years or older who attended or were admitted to the Department of Gastroenterology at Yokohama City University (YCU) Hospital between November 2020 and April 2022. Those who were taking antimicrobials within one month prior to periodontal examination and those with edentulous jaws were excluded from the study. This study was approved by the research ethics committee of Kanagawa Dental University (KDU) and YCU and was conducted at YCU Hospital in compliance with the Declaration of Helsinki. All the participants were informed of the purpose, outline, safety, and protection of personal information of this study, and their written consent to participate in the study based on their free will was obtained. Initially, sixty-nine participants were enrolled, and data from sixty participants (forty-one with MASH and nineteen with MASH-HCC) for whom all testing and sample collection data were available were used for the analysis.

### 2.2. Background Information

The participants’ gender, age, and smoking status were interviewed using a questionnaire. The dentist filled out the response form based on the participants’ responses. Body mass index was calculated from the medical records by obtaining the height and weight values closest to the date of periodontal examination.

### 2.3. Periodontal Examination

Periodontal examinations were performed by two dentists from the Department of Periodontology at KDU. The probing depth and bleeding on probing were measured at the six probing points per tooth. The plaque index was recorded at the four points per tooth, and tooth mobility was evaluated. Probing was performed at a constant pressure using a plastic probe (Contact Probe, Nihon Dental Laboratory Co., Ltd., Tokyo, Japan) with a probing pressure of 0.2 N. The dentists calibrated their probing tools in advance. A periodontal jaw model (P15FE-500HPRO-S2A1-GSF, Nissin, Kyoto, Japan) was used for calibration.

### 2.4. Sample Collection

Saliva samples used for the IgA concentration assay were collected using the Salivette^®^ (SARSTEDT, Nümbrecht, Germany). A sponge made of polypropylene–polyethylene polymer was held under the tongue for 2 min, and the saliva-containing sponge was returned to the tube. The tubes were quickly ice-cooled, centrifuged (1500× *g*, 20 min, 4 °C), and stored at –80 °C until analysis. Mouth-rinsed water was used to analyze the oral microbiota. The saliva used for the bacterial flora analysis was collected by having the participant gargle 7.5 mL of physiological saline for 10 s to mix with the saliva, and collecting the fluid spat out by the participant in a tube. The tubes were immediately ice-cooled, frozen at −80 °C, and sent for the bacterial flora analysis to the Medical Laboratory (Cykinso, Inc., Tokyo, Japan). Fecal samples were collected from the participants using a Mykinso fecal collection kit^®^ (Cykinso, Inc.) containing a guanidine thiocyanate solution. The fecal samples were collected by the participants themselves according to the manufacturer’s manual. The samples were immediately transported at room temperature to the Medical Laboratory (Cykinso, Inc.).

### 2.5. Medical Examination

Peripheral blood samples were collected on the same day as periodontal examination. Endotoxin, high-sensitivity C-reactive protein (CRP), aspartate transaminase (AST), alanine aminotransferase (ALT), and total bilirubin (T-Bil) levels were analyzed.

### 2.6. Assay of IgA Concentration in Saliva

IgA concentrations in saliva were determined by the enzyme-linked immunosorbent assay (ELISA) using a Human IgA ELISA Kit (Bethyl Laboratories, Inc., Montgomery, TX, USA). The ELISA was performed according to the manufacturer’s instructions.

### 2.7. DNA Extraction

DNA extraction from the saliva and fecal samples was performed at the Medical Laboratory (Cykinso, Inc.) using an automated DNA extraction machine (GENE PREP STAR PI-1200A, Kurabo Industries Ltd., Osaka, Japan) according to the manufacturer’s protocol. The V1–V2 region of the 16S rRNA gene was amplified using the forward primer 16S_27Fmod(TCG TCG GCA GCG TCA GAT GTG TAT AAG AGA CAG AGR GTT TGA TYM TGG CTC AG) and reverse primer 16S_338R (GTC TCG TGG GCT CGG AGA TGT GTA TAA GAG ACA GTG CTG CCT CCC GTA GGA GT) with the KAPA HiFi Hot Start Ready Mix (Roche, Basel, Switzerland). To sequence 16S amplicons on the Illumina MiSeq platform, dual index adapters were attached using the Nextera XT Index kit. Each library was diluted to 5 ng/µL, and equal volumes of the libraries were mixed to 4 nM. The DNA concentration of the mixed libraries was quantified using qPCR with the KAPA SYBR FAST qPCR Master mix (KK4601, KAPA Biosystems, Inc., Wilmington, MA, USA) along with primer 1 (AAT GAT ACG GCG ACC ACC) and primer 2 (CAA GCA GAA GAC GGC ATA CGA). Library preparation was carried out according to the 16S library preparation protocol of Illumina (Illumina, San Diego, CA, USA). The libraries were sequenced using the MiSeq Reagent Kit v2 (500 Cycles) in the 250 bp paired-end mode.

### 2.8. Taxonomy Assignment Based on 16S rRNA Gene Sequences

The paired-end reads of the partial 16S rRNA gene sequences were analyzed using QIIME 2 (version 2020.8). The steps for data processing and assignment based on the QIIME 2 pipeline were as follows: (1) using DADA2 for joining the paired-end reads, filtering, and denoising; (2) assigning taxonomic information to each ASV using the naive Bayes classifier in QIIME 2 based on the 16S gene (V1–V2 region) data from SILVA (version 138) to determine the identity and composition of the bacterial genera.

### 2.9. Bayesian Network Analysis and Classification Trees

A Bayesian network is a directed acyclic graph composed of a set of variables {X1, X2, …, XN} and a set of directed edges between them [16]. The details of the analytical methods are described in our previous report [17]. Because the Bayesian network could not be analyzed with missing values, we excluded one participant from the MASH group who had missing T-Bil values, and data from fifty-nine participants (forty patients with MASH and nineteen patients with MASH-HCC) were used for the analysis. Based on the results of Bayesian network analysis, a classification tree analysis was performed using rpart.

### 2.10. Statistical Analysis

Statistical analyses were performed using SPSS Statistics (version 27.0; IBM, New York, NY, USA) and R (version 3.5.1 (The R Project for Statistical Computing, Vienna, Austria, 2018). The Mann–Whitney U test was used for comparisons between the two groups, except for gender and smoking status, which were verified by the χ^2^ test. Spearman’s rank correlation coefficient was used for the correlation analysis. Statistical significance was set at *p* < 0.05.

## 3. Results

### 3.1. Participant’s Information, Periodontal and Medical Examinations, and Salivary IgA Levels

Table 1 shows the information on the participants, periodontal and medical status, and IgA concentration in the saliva (the data are also reported in our previous study [15]). Compared with the MASH group, the MASH-HCC group was significantly older (*p* = 0.0004). Both groups showed similar periodontal examination results. The salivary IgA concentration was significantly lower in the MASH-HCC group than in the MASH group (*p* < 0.001). The endotoxin and T-Bil levels were significantly higher in the MASH-HCC group (*p* < 0.0001) than in the MASH group (*p* = 0.014).

### 3.2. Diversity and Composition of Bacterial Flora in the Saliva

**Figure 1:**
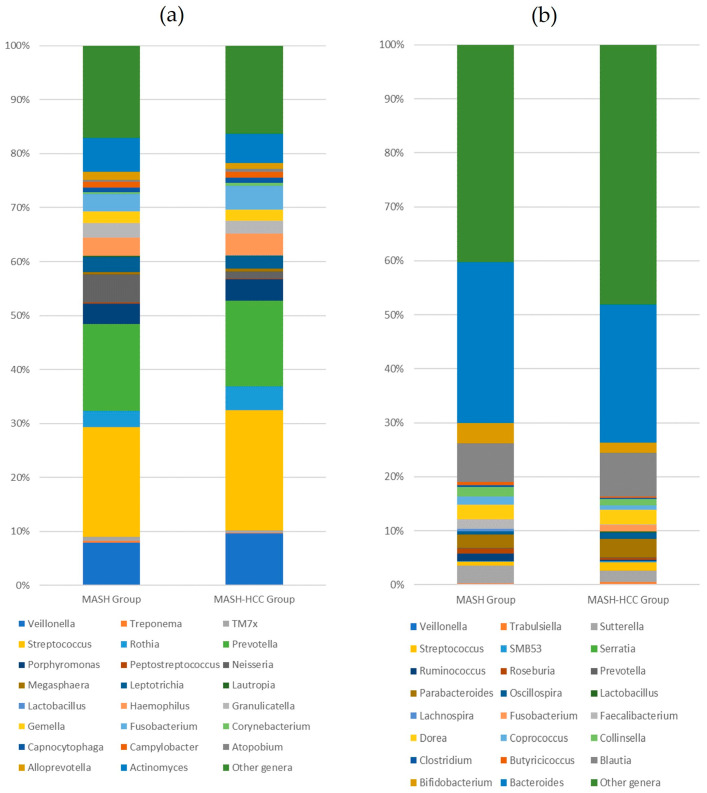
Visualization of the microflora sequencing results in the MASH and MASH-HCC groups using 100% stacked bar charts. (**a**) The bacterial composition of the salivary bacterial genera. (**b**) The bacterial composition of the fecal bacterial genera.

Table 2 shows the diversity and abundance of the salivary microbiome. All the bacterial phyla detected, 23 bacterial genera with an average occupancy greater than 0.5%, and 22 bacterial species with an average occupancy greater than 0.1% are presented. The Shannon index of the salivary microflora was significantly lower in the MASH-HCC group than in the MASH group (*p* = 0.03). The observed operational taxonomic units (OTUs) were comparable between the two groups. Regardless of the liver disease type, the predominant microorganisms at the phylum level in all the participants were *Bacillota*, *Bacteroidota*, *Pseudomonadota*, *Actinomycetota*, and *Fusobacteriota*. Similarly, the genera *Streptococcus*, *Prevotella*, *Veillonella*, and *Actinomyces* predominated in both groups. Their occupancy rate was not significantly different between the two groups. The compositions of the salivary bacterial genera are visualized in Figure 1a.

The proportions of *P. gingivalis* and *F. nucleatum* were higher in the MASH-HCC group than in the MASH group. However, only *F. nucleatum* showed a significant difference (*p* = 0.014). Conversely, the occupancy rate of *Treponema denticola* was significantly lower in the MASH-HCC group than in the MASH group (*p* = 0.02).

### 3.3. Diversity and Composition of Bacterial Flora in the Feces

Table 3 shows the diversity and abundance of bacterial flora in feces, including all the bacterial phyla detected and 23 bacterial genera with top occupancy. The Shannon index was significantly lower in the MASH-HCC group than in the MASH group (*p* < 0.001). The observed OTUs were comparable between the two groups. At the phylum level, both groups were dominated by *Bacillota*, *Bacteroidota*, *Actinomycetota,* and *Pseudomonadota.* However, the occupancy rates were comparable between the two groups. Only *Fusobacteriota* was significantly more prevalent in the MASH-HCC group than in the MASH group (*p* = 0.002).

At the genus level, *Bacteroides* and *Blautia* dominated all the participants, but their occupancy rates were comparable between the two groups. The occupancy rates of *Butyricicoccus* (*p* = 0.022) and *Roseburia* (*p* < 0.05) in the MASH-HCC group were significantly lower than those in the MASH group. The proportion of *Faecalibacterium* in the MASH-HCC group was approximately one-fourteenth of that in the MASH group. Conversely, the occupancy rates of *Fusobacterium* and *Lactobacillus* in the MASH-HCC group were higher than in the MASH group; however, only *Fusobacterium* showed a significant difference (*p* = 0.002). The compositions of the fecal bacterial genera are visualized in Figure 1b.

### 3.4. Determination of Causal Effects Using Bayesian Network Analysis

**Figure 2:**
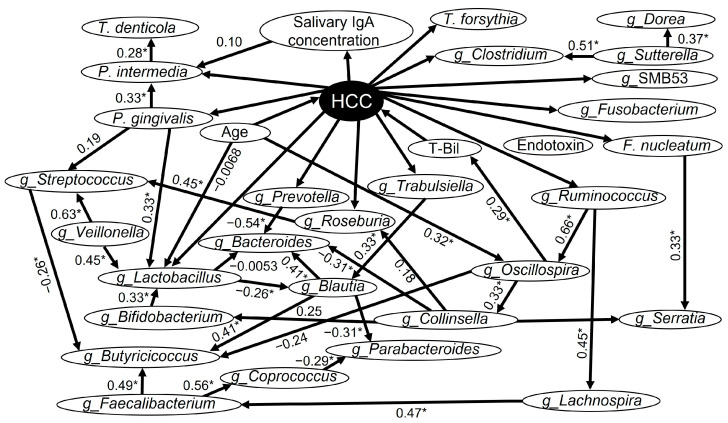
Bayesian network with the graphical representation of the causal relationships between the factors. The analyzed factors consisted of a total of 32 items (4 items that showed significant differences in Table 1, 5 oral periodontal bacterial species, and 23 genera of top occupying intestinal bacteria). g_: enterobacterial genera; HCC: hepatocellular carcinoma. The source of the arrow is the cause and the destination is the effect. The numbers listed on the side of the arrows are Spearman’s rank correlation coefficients (*n* = 59). Statistical superiority was defined as *p* < 0.05, in which case the numbers were marked with an *.

Figure 2 shows the results of the Bayesian network analysis. We focused on the items that showed significant differences in the comparison between the two groups (Table 1, Table 2 and Table 3) and added the major oral periodontopathic bacterial species and the top 23 enterobacterial genera (Table 3), setting them as factors in the Bayesian network analysis.

The presence of HCC directly affected the following major periodontal bacterial species: *P. gingivalis*, *Tannerella forsythia*, *F. nucleatum*, and *Prevotella intermedia* in the saliva. It also affected the salivary IgA concentrations. Furthermore, the salivary IgA concentrations affected *P. intermedia* in the saliva.

HCC also directly affected the genera *Lactobacillus, Roseburia, Fusobacterium, Prevotella*, *Clostridium*, *Ruminococcus*, *Trabulsiella,* and SMB53 in the feces. In addition, *P. gingivalis* in the saliva directly affected *Lactobacillus* and *Streptococcus* in the feces and indirectly affected *Blautia* and *Butyricicoccus.* Moreover, salivary *F. nucleatum* affected *Serratia* in the feces. Meanwhile, the T-Bil level and age had a direct impact on HCC. The genus *Oscillospira* in the feces affected T-Bil.

### 3.5. Classification Tree to Assess Disease Type

**Figure 3:**
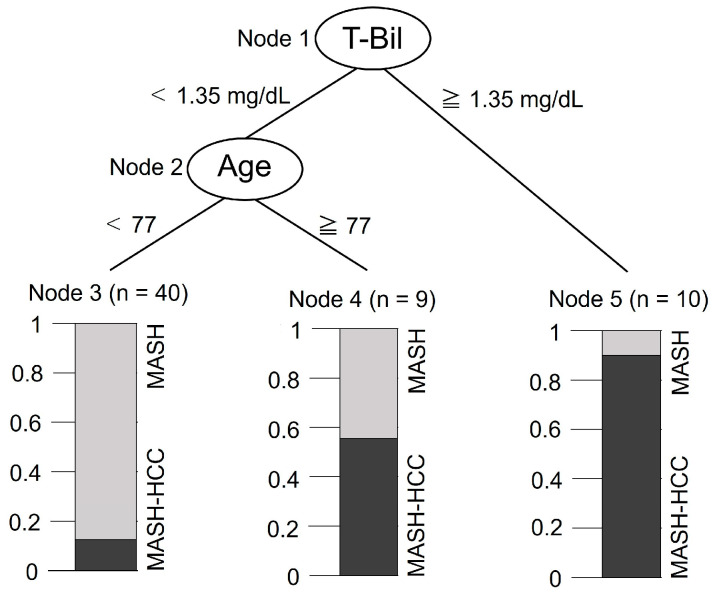
Validated classification tree with hepatocellular carcinoma (HCC) as the dependent variable and the total bilirubin (T-Bil) and age as the explanatory variables.

The Bayesian network results showed that the two factors affecting HCC were the T-Bil level and age. When the dependent variable was set as the presence or absence of HCC and the explanatory variables were set as the T-Bil and age in a classification tree (Figure 3), the major factor affecting HCC was the T-Bil, followed by age. HCC develops when the T-Bil exceeds 1.35 mg/dL in the MASH. However, even if the T-Bil is less than 1.35 in MASH, HCC develops when the patient is over 77 years of age.

## 4. Discussion

This is the first study to analyze the relationship between oral periodontal pathogenic bacteria and intestinal bacteria in patients with MASH and MASH-HCC. In this study, the presence of HCC directly affected several periodontopathogenic bacteria in the saliva. In addition, a higher abundance of *F. nucleatum* in the saliva was observed in the MASH-HCC group than in the MASH group. It has been reported that the oral bacterial flora changes in patients with pancreatic cancer [18], and the presence of *F. nucleatum* in the oral cavity is elevated in patients with lung and colorectal cancers [19,20]. Cancer occurs when the systemic immune response decreases [21], and immune function clearly decreases in patients with cancer [22]. In addition, since the type and number of oral bacteria are related to systemic immune status [23], in this study, the decline in systemic immune function caused by MASH-HCC may have affected periodontal bacteria in the oral cavity, which is a remote organ.

The abundance of salivary *F. nucleatum* in the MASH-HCC group was higher than that of the other periodontal pathogenic bacteria, except for *P. gingivalis*. *F. nucleatum* is an opportunistic bacterium present in the oral cavity of individuals without periodontal disease [24]. In addition, Leigh et al. [25] reported that opportunistic bacteria in the oral cavity increase owing to a decline in immune function. Hence, although there was no difference in the periodontal conditions between the two groups in this study, the MASH-HCC group had decreased systemic immune function; therefore, the occupancy rate of *F. nucleatum* in the oral cavity may have been high.

The Bayesian network analysis revealed that HCC directly affected several fecal bacteria; however, none of the fecal bacteria directly affected HCC. Among the intestinal bacteria directly affected by HCC, the genera *Roseburia* and *Faecalibacterium* showed lower abundances in the MASH-HCC group than in the MASH group, whereas the genus *Fusobacterium* had a higher abundance. Various studies have reported that gastrointestinal cancer is associated with the intestinal microbiome. The genus *Roseburia* was decreased in the intestinal microbiota of patients with colorectal and pancreatic cancer [26,27]. Although there is a known case of MASH that developed into MASH-HCC through liver cirrhosis [28], *Roseburia* occupancy was decreased in the intestinal microbiota of patients with liver cirrhosis [29]. A decreased occupancy of the genus *Faecalibacterium* has been reported in patients with hepatocellular carcinoma [30]. The abundance of the genus *Fusobacterium* increases in the feces of patients with colorectal cancer [31,32]. The abundance of *Fusobacterium* increases and that of *Roseburia* decreases when dysbiosis occurs in the intestinal microbiota [33]. The liver and intestinal microbiomes have a close bidirectional relationship [34], and dysbiosis occurs in patients with liver cancer [35]. Moreover, the Shannon index, which shows the diversity of the intestinal microflora, decreases owing to dysbiosis [36]. The Shannon index was lower in the MASH-HCC group than in the MASH group in this study. Hence, it is likely that the MASH-HCC group in the present study had more advanced dysbiosis.

High-fat, high-glucose, and low-fiber Western diets are known to accelerate progression from MASH to MASH-HCC [37]. In addition, the Western diet increases the abundance of the genus *Fusobacterium* and decreases the abundance of the genus *Roseburia* in the intestine [38]. Thus, high-fat, high-glucose, and low-fiber diets that cause HCC may also affect the intestinal bacteria. In the present Bayesian network analysis, the result that HCC had a direct effect on the fecal bacteria may be attributed to the high-fat, high-glucose, and low-fiber diets consumed for long periods by the patients with MASH-HCC, which directly affect intestinal bacteria.

Our results showed that the genus *Fusobacterium* in the feces did not affect HCC, and *F. nucleatum* in the saliva did not affect the genus *Fusobacterium* in the feces. Guo et al. reported that *F. nucleatum* is increased in hepatocellular carcinoma tissues and that hepatocellular carcinoma is affected by *F. nucleatum* because methyltransferase-like protein 3 expression during *F. nucleatum* infection is involved in tumor progression [39]. Although not revealed in the present study, intestinal bacteria may affect HCC.

Primary bile acids increase in MASH-HCC, and *Lactobacillus*, which metabolizes them, has been reported to increase in the intestine [40]. Therefore, the Bayesian network analysis revealed a direct effect of MASH-HCC on *Lactobacillus* spp. in the feces. Furthermore, the occupancy rate of *Lactobacillus* spp. in the feces of the MASH-HCC group was higher than that in the MASH group. Interestingly, not only HCC but also *P. gingivalis* in the saliva directly affected the genus *Lactobacillus* in the feces. *P. gingivalis* is a typical periodontal pathogenic bacterium in the oral cavity that can affect intestinal bacteria and cause dysbiosis [41]. Park et al. [42] found that mice infected with *P. gingivalis* in the oral cavity showed increased levels of the intestinal phyla *Actinobacteria* and *Deferribacteres*. In addition, Nakajima et al. [43] reported that a single oral dose of *P. gingivalis* administered to mice increased *Bacteroidetes* and decreased *Firmicutes* in the intestine. The oral administration of *P. gingivalis* causes changes in the intestinal microbiota, impairs intestinal barrier function, and damages the liver [43]. Although the difference was not significant, the occupancy rate of salivary *P. gingivalis* was higher in the MASH-HCC group. Hence, in addition to HCC, the high abundance of *P. gingivalis* in saliva could have damaged the liver and altered the amount of primary bile acids, which could have affected the genus *Lactobacillus*.

We found that *P. gingivalis* in the saliva had a direct effect on the genus *Streptococcus* in the feces. In patients with atrophic gastritis in the gastric corpus who are at a high risk of gastric cancer, an increase in *Streptococcus* spp. in the stomach [44] and in the feces of patients with colorectal cancer [45] has been reported, and the genus *Streptococcus* is associated with digestive disorders. Thus, in the MASH-HCC group, an increase in *P. gingivalis* in the oral cavity can cause dysbiosis in the intestine, which may have affected *Streptococcus* spp.

The causal analysis indicated that salivary *P. gingivalis* caused a decrease in *Blautia* and *Bacteroides* via the genus *Lactobacillus* and *Butyricicoccus* via the genus *Streptococcus*. The dysbiosis caused by the increased *P. gingivalis* in the oral cavity would have resulted in an increase in the genera *Lactobacillus* and *Streptococccus* and a consequent decrease in the genera *Blautia*, *Bacteroides*, and *Butyricoccus* in the gut. Studies have shown an association between these three intestinal bacteria and gastrointestinal cancer. *Blautia* spp. are decreased in the feces of liver cancer patients [46], *Bacteroides* spp. are decreased in the feces of mice that developed liver cancer due to a high-fat, high-cholesterol diet [47], and *Butyricicoccus* spp. are decreased in the feces of patients with esophageal cancer [48]. All of these bacterial genera are short-chain fatty acid (SCFA) producers [49,50,51]. McBrearty et al. [52] reported that since SCFAs have strong anti-inflammatory and anti-tumor effects, the administration of SCFA to mice delayed the development of hepatocellular carcinoma. These bacterial genera did not directly affect HCC but may have affected HCC via SCFA. Hence, reducing *P. gingivalis* in the oral cavity, which indirectly affects these three intestinal bacteria, may help prevent the development of MASH-HCC.

Our data demonstrated that salivary *F. nucleatum* affected the fecal *Serratia* spp., which is an opportunistic bacterium like *F. nucleatum* [23,53]. Lin et al. [54] reported increased levels of both oral *F. nucleatum* and gut opportunistic bacteria in a mouse model of ulcerative colitis. In the present study, the patients with MASH-HCC would have had a generalized state of weakened immune system that made them susceptible to an increase in both the opportunistic bacteria *F. nucleatum* and the genus *Serratia*. Therefore, our results show that *F. nucleatum* in the oral cavity directly affects *Serratia.*

Interestingly, the salivary IgA concentrations only affected *P. intermedia* in the saliva. Salivary IgA levels increase with the number of periodontal pathogenic bacteria and control them [55,56,57]. Despite this, the fact that the salivary IgA concentration only affected *P. intermedia* in this study suggests that the effect of HCC on periodontal pathogenic bacteria in the oral cavity was greater than that of the salivary IgA concentration.

Of the two factors directly affecting HCC, one was the blood T-Bil level, which was directly affected by fecal *Oscillospira* spp. T-Bil levels increase as liver function declines in patients with liver cancer [58]. Increased T-Bil levels have also been reported in rats with liver cancer [59]. Therefore, it is likely that the MASH-HCC group in this study showed a decline in liver function, resulting in high T-Bil levels. Furthermore, an increase in the secondary bile acids produced by intestinal bacteria decreases liver function [60], and the level of secondary bile acids in feces is positively correlated with genus *Oscillospira* in feces [61]. The genus *Oscillospira* may have affected the increase in the T-Bil levels by reducing liver function through the secondary bile acids.

Age is another factor that directly affects HCC development. The median age of the patients in the MASH-HCC group was higher than that in the MASH group. Recently, Shimomura et al. [62] reported that patients with MASH-HCC were older and had lower antioxidant function than patients with MASH and that oxidative stress correlated with MASH activation markers, both of which were increased. However, young patients had lower levels of MASH activation markers because their antioxidant functions were preserved [62]. Hence, old age may be a major risk factor for MASH development.

The results of the classification tree analysis suggested that a T-Bil of 1.35 mg/dL or higher in the MASH group was related to the occurrence of HCC. HCC also occurs at the age of 77 years or older, even when T-Bil is less than 1.35 mg/dL in MASH. Therefore, MASH patients with a T-Bil of 1.35 mg/dL or higher, or older MASH patients with T-Bil less than 1.35 mg/dL may require more medical assistance to prevent them from developing HCC. High T-Bil levels are the major factors affecting HCC. The genus *Oscillospira*, which elevates T-Bil levels, increases when dysbiosis occurs in the intestines [63]. Because *P. gingivalis* in the oral cavity causes intestinal dysbiosis [43], patients with MASH may require periodontal management to suppress the abundance of *P. gingivalis* in the oral cavity to prevent dysbiosis.

As our Bayesian network analysis showed that HCC had a direct effect on periodontopathogenic bacteria and fecal bacteria, we focused on the effect of HCC on intestinal bacteria. In recent years, the “gut–liver axis” mechanism has been identified, and it is now clear that the gut and liver are closely related to each other [64]. Thus, HCC and intestinal bacteria may interact with each other. The genera *Roseburia* and *Faecalibacteria*, found to be decreased in the feces of the MASH-HCC group, are butyrate-producing bacteria [65,66]. The intestinal bacteria *Blautia*, *Bacteroides*, and *Butyricicoccus*, which are indirectly affected by *P. gingivalis* in saliva, are also SCFA-producing bacteria [49,50,51]. Hence, the development of MASH to MASH-HCC may be related to the SCFAs produced by intestinal bacteria.

Our causal analysis revealed a direct effect of MASH-HCC on *Lactobacillus* spp. in feces. Furthermore, the occupancy of *Lactobacillus* spp. in the feces of the MASH-HCC group was higher than that in the MASH group. Contrary to our results in the present study, *Lactobacillus* spp. are reported to be decreased in the intestines of patients with MASH-HCC [67]. As *Lactobacillus* spp. have also been reported to exert anti-tumor effects [67], further research on the relationship between MASH-HCC and *Lactobacilli* is warranted.

### Limitations

The current study has several limitations. First, the number of participants was low. This is because it was difficult to recruit participants who met the inclusion criteria. Therefore, the number of participants in the two groups could not be matched. Second, differences were observed in the ages of the participants in the MASH and MASH-HCC groups. Future studies will need to set the age of the participants higher in order to keep the age of both groups the same. In this study, we aimed to determine the causal relationship between oral periodontal bacteria and intestinal bacteria in the development of MASH-HCC. Thus, we did not determine the similarity between the oral and intestinal bacteria of the patients with MASH and those with MASH-HCC. An analysis such as the PERMANOVA-S method [68] is needed to clarify the similarity between the oral and intestinal bacteria.

## 5. Conclusions

MASH-HCC directly affects periodontal pathogenic bacteria, salivary IgA, and intestinal bacteria. *P. gingivalis* may, directly and indirectly, affect the intestinal bacteria associated with gastrointestinal cancer.

## Figures and Tables

**Table 1:** Demographic factors, periodontal and medical conditions, and salivary IgA levels.

Parameter	MASH Group (N = 41)	MASH-HCC Group (N = 19)	*p*-Value
Gender (men/women)	25/16	13/6	0.578
Smoking status (+/−)	9/32	4/15	0.973
Age (years)	59 (55–70)	79 (64–82)	0.0004 *
BMI	26.2 (22.3–31.5)	27.7 (25.9–30.8)	0.503
Number of remaining teeth	26 (21–27)	25 (21–27)	0.598
PD (mm)	2.8 (2.6–3.2)	2.9 (2.6–3.2)	0.653
BOP (%)	14.9 (10.7–24.1)	15.4 (7.3–31.4)	0.799
Tooth mobility	0 (0–0.1)	0 (0–0.1)	0.703
PlI	0.9 (0.8–1.4)	0.9 (0.7–1.4)	0.619
Salivary IgA (ug/mL)	231.7 (146.5–482.8)	102.7 (85.8–168.9)	<0.001 *
Endotoxin (EU)	0.13 (0.08–0.17)	0.22 (0.15–0.28)	<0.0001 *
CRP (mg/dL)	0.14 (0.09–0.48)	0.13 (0.07–0.34)	0.487
AST (U/L)	51 (27–62)	38 (29–58)	0.546
ALT (U/L)	53 (26–71)	29 (23–44)	0.094
T-Bil (mg/dL)	0.8 (0.6–1)	1.3 (0.7–1.7)	0.014 *

BMI, body mass index; PD, probing depth; BOP, bleeding on probing; PlI, plaque index; CRP, C-reactive protein; AST, aspartate aminotransferase; ALT, alanine aminotransferase; T-Bil, total bilirubin. BOP (+) = 1; BOP (−) = 0. The figures for gender and smoking status indicate the number of individuals, and statistical analysis was performed by the χ^2^ test. The presented values for the other items are medians (first quartile–third quartile), and the Mann–Whitney U test was used for the statistical analysis (* *p* < 0.05).

**Table 2:** Bacterial phyla, genera, and species of the salivary microbiota.

Parameter	MASH Group (N = 41)	MASH-HCC Group (N = 19)	*p*-Value
Diversity of bacterial flora			
Shannon index	6.72 (6.45–7.13)	6.51 (6.05–6.80)	0.03 *
Observed OTUs	257 (207–296)	230 (205–280)	0.119
Phylum			
*Actinomycetota* (%)	11.7 (9.56–15.19)	13.15 (10.38–14.25)	0.661
*Bacillota* (%)	39.5 (36.94–43.06)	42.7 (35.56–45.30)	0.604
*Bacteroidota* (%)	24.94 (21.61–28.02)	23.45 (20.54–30.08)	0.450
*Campylobacterota* (%)	0.976 (0.618–1.50)	1.13 (0.806–1.57)	0.418
*Cyanobacteria* (%)	0 (0–0)	0 (0–0)	0.928
*Desulfobacterota* (%)	0 (0–0.0346)	0.0135 (0–0.0208)	0.945
*Fusobacteriota* (%)	8.12 (3.94–9.81)	8.23 (5.42–9.61)	0.335
*Patescibacteria (%)*	1.83 (0.463–2.54)	1.32 (0.337–2.01)	0.167
*Pseudomonadota* (%)	13.93 (6.79–16.71)	13.17 (3.01–17.39)	0.727
*Spirochaetota* (%)	0.269 (0.025–0.638)	0.061 (0.029–0.386)	0.185
*Synergistetes* (%)	0.040 (0–0.103)	0.020 (0.013–0.025)	0.228
Genus			
*Actinomyces* (%)	6.27 (3.62–8.05)	5.39 (3.84–6.72)	0.418
*Alloprevotella* (%)	1.54 (0.788–3.26)	1.16 (0.691–1.91)	0.348
*Atopobium* (%)	0.337 (0.165–0.774)	0.495 (0.147–0.768)	0.962
*Campylobacter* (%)	1.02 (0.650–1.50)	1.13 (0.806–1.57)	0.525
*Capnocytophaga* (%)	0.897 (0.282–1.85)	0.976 (0.524–1.70)	0.391
*Corynebacterium* (%)	0.306 (0.191–0.804)	0.484 (0.146–0.931)	0.340
*Fusobacterium* (%)	3.25 (1.84–5.07)	4.48 (2.24–5.58)	0.266
*Gemella* (%)	2.15 (1.14–3.07)	2.04 (1.17–3.29)	0.949
*Granulicatella* (%)	2.73 (1.86–3.73)	2.39 (1.60–3.16)	0.221
*Haemophilus* (%)	3.36 (1.70–5.34)	3.96 (1.10–5.77)	0.836
*Lactobacillus* (%)	0.00620 (0–0.239)	0.122 (0–1.02)	0.247
*Lautropia* (%)	0.141 (0–0.633)	0.138 (0–0.831)	0.572
*Leptotrichia* (%)	2.83 (1.36–4.58)	2.23 (1.53–3.84)	0.567
*Megasphaera* (%)	0.413 (0.151–0.873)	0.536 (0.106–1.03)	0.861
*Neisseria* (%)	5.20 (1.14–8.72)	1.25 (1.03–9.20)	0.505
*Peptostreptococcus* (%)	0.256 (0.0694–0.643)	0.183 (0–0.664)	0.262
*Porphyromonas* (%)	3.71 (1.72–7.14)	3.98 (0.900–6.49)	0.589
*Prevotella* (%)	16.1 (11.8–19.3)	15.9 (11.6–20.6)	0.799
*Rothia* (%)	3.09 (1.53–5.53)	4.42 (2.38–5.42)	0.409
*Streptococcus* (%)	20.3 (16.1–24.6)	22.2 (16.0–27.0)	0.340
TM7x (%)	0.729 (0.221–1.509)	0.463 (0.0127–1.04)	0.249
*Treponema* (%)	0.292 (0.0331–0.561)	0.0611 (0.0302–0.225)	0.0768
*Veillonella* (%)	7.98 (6.07–10.7)	9.68 (6.30–12.4)	0.204
Species			
*Actinomyces israelii* (%)	0 (0–0)	0 (0–0.0256)	0.101
*Bifidobacterium dentium* (%)	0 (0–0.040)	0 (0–0.0748)	0.620
*Capnocytophaga gingivalis* (%)	0.345 (0.130–0.691)	0.427 (0.154–1.25)	0.193
*Dialister pneumosintes* (%)	0 (0–0.0821)	0 (0–0.0587)	0.917
*Fusobacterium nucleatum* (%)	0.189 (0.0200–0.443)	0.362 (0.170–0.928)	0.014 *
*Lactobacillus crispatus* (%)	0 (0–0)	0 (0–0.00302)	0.544
*Lactobacillus fermentum* (%)	0 (0–0.287)	0 (0–0.150)	0.661
*Lactobacillus gasseri* (%)	0 (0–0.0257)	0 (0–0.122)	0.165
*Lactobacillus salivarius* (%)	0 (0–0.00405)	0 (0–0.0874)	0.363
*Metamycoplasma hyosynoviae* (%)	0 (0–0.0576)	0 (0–0.0599)	1.000
*Porphyromonas endodontalis* (%)	0.457 (0.138–0.982)	0.195 (0–0.377)	0.0923
*Porphyromonas gingivalis* (%)	0.138 (0–1.26)	0.400 (0–0.725)	0.520
*Prevotella denticola* (%)	0.459 (0.0750–0.948)	0.429 (0.0683–0.924)	0.905
*Prevotella enoeca* (%)	0 (0–0.00780)	0 (0–0)	0.149
*Prevotella intermedia* (%)	0 (0–0.334)	0 (0–0.158)	0.532
*Prevotella nigrescens* (%)	0 (0–0.0243)	0 (0–0)	0.175
*Streptococcus anginosus* (%)	0.124 (0.0379–0.247)	0.165 (0.0126–0.373)	0.937
*Streptococcus mutans* (%)	0.0110 (0–0.0608)	0.0685 (0–0.118)	0.159
*Streptococcus pneumoniae* (%)	0.557 (0.277–0.998)	0.376 (0–1.43)	0.260
*Streptococcus sobrinus* (%)	0 (0–0.0183)	0 (0–0.00838)	0.919
*Tannerella forsythia* (%)	0.146 (0.0612–0.287)	0.0827 (0.0200–0.328)	0.661
*Treponema denticola* (%)	0.0638 (0–0.195)	0 (0–0.0325)	0.0223 *

OTUs, operational taxonomic units. The values are presented as medians (first quartile–third quartile), and the Mann–Whitney U test was used for the statistical analysis (* *p* < 0.05).

**Table 3:** Bacterial phyla and genera of the fecal microbiota.

Parameter	MASH Group (N = 41)	MASH-HCC Group (N = 19)	*p*-Value
Diversity of bacterial flora			
Shannon index	5.79 (5.54–6.17)	5.43 (4.92–5.56)	<0.001 *
Observed OTUs	173 (143–244)	168 (115–265)	0.886
Phylum			
*Actinomycetota* (%)	5.92 (2.54–9.96)	4.62 (1.91–9.57)	0.515
*Bacillota* (%)	46.8 (42.1–51.1)	45.9 (42.1–51.7)	0.793
*Bacteroidota (%)*	37.3 (31.5–42.6)	35.7 (31.2–41.2)	0.634
*Campylobacterota* (%)	0 (0–0)	0 (0–0)	0.307
*Desulfobacterota* (%)	0.210 (0.0138–0.687)	0.203 (0–0.753)	0.719
*Fusobacteriota* (%)	0.0172 (0–0.452)	1.11 (0.263–2.32)	0.002 *
*Patescibacteria* (%)	0 (0–0)	0 (0–0)	0.580
*Pseudomonadota* (%)	4.98 (3.99–10.6)	6.11 (3.46–12.3)	0.861
*Spirochaetota* (%)	0 (0–0)	0 (0–0)	0.197
*Synergistetes* (%)	0 (0–0)	0 (0–0)	1.000
*Verrucomicrobia* (%)	0 (0–0)	0 (0–0)	0.776
Genus			
*Bacteroides* (%)	29.8 (19.0–34.5)	25.6 (19.4–29.4)	0.360
*Bifidobacterium* (%)	3.84 (0.39–9.48)	1.87 (1.02–7.72)	0.836
*Blautia* (%)	7.16 (4.66–10.8)	8.11 (1.31–10.4)	0.424
*Butyricicoccus* (%)	0.540 (0.195–0.825)	0.170 (0.029–0.370)	0.022 *
*Clostridium* (%)	0.310 (0.165–0.820)	0.270 (0.130–0.770)	0.594
*Collinsella* (%)	1.83 (0.0100–2.98)	1.20 (0.0100–3.00)	0.930
*Coprococcus* (%)	1.51 (0.375–4.17)	0.830 (0.140–2.00)	0.133
*Dorea* (%)	2.70 (1.71–4.62)	2.68 (0.460–4.05)	0.259
*Faecalibacterium* (%)	1.68 (0–6.16)	0.119 (0–3.45)	0.282
*Fusobacterium* (%)	0.0100 (0–0.550)	1.11 (0.260–2.00)	0.002 *
*Lachnospira* (%)	0.479 (0.0150–1.65)	0.070 (0.0100–1.00)	0.292
*Lactobacillus* (%)	0.0200 (0–0.505)	0.220 (0–8.54)	0.104
*Oscillospira* (%)	0.610 (0.205–1.43)	1.19 (0.290–1.75)	0.500
*Parabacteroides* (%)	2.51 (1.26–5.93)	3.42 (1.73–5.83)	0.490
*Prevotella* (%)	0.0100 (0–0.0300)	0.0200 (0.0100–0.850)	0.074
*Roseburia* (%)	0.970 (0.380–1.76)	0.360 (0.150–1.29)	<0.05 *
*Ruminococcus* (%)	1.38 (0.105–4.00)	0.239 (0.110–1.80)	0.294
*Serratia* (%)	0.0200 (0–0.560)	0.0100 (0–4.36)	0.562
SMB53 (%)	0.110 (0.0200–0.265)	0.239 (0–0.650)	0.707
*Streptococcus* (%)	0.790 (0.190–3.45)	1.56 (0.210–4.42)	0.634
*Sutterella* (%)	3.20 (0.985–4.12)	2.13 (0.580–3.87)	0.259
*Trabulsiella* (%)	0.270 (0.040–0.920)	0.440 (0.090–2.39)	0.499
*Veillonella* (%)	0.0500 (0–1.23)	0.050 (0.0100–0.650)	0.797

OTUs, operational taxonomic units. The values are presented as medians (first quartile–third quartile), and the Mann–Whitney U test was used for the statistical analysis (* *p* < 0.05).

## Data Availability

All the data obtained in this study are described in the revised manuscript.
